# The Growth of the Nursing Profession: The Habit of Talking Too Much

**Published:** 1918-01-19

**Authors:** 


### THE MATRONS' AND SISTERS' DEPARTMENT.

### THE GROWTH OF THE NURSING PROFESSION.

### I. The Habit of Talking Too Much.

THE general impression made upon the mind of the average reader of the morning papers is that everyone is talking too much, and it is possible that this might likewise be the impression of the average reader of the nursing periodicals. The thoughtful mind would also be struck by the apparent facility with which black can be made to appear white, and white black. There is one point, however, upon which all seem to be agreed, and that is, that nursing and all things connected therewith are in the melting-pot. It seems to us that it might be useful at the present stage to see exactly what it is that we are thus happily consigning to the melting-pot, why we are doing it, and what we expect or desire to result from this process of liquefaction.

The words, "the Nursing Profession," have been hard-worked of late; some assert that only within the last ten years has nursing come into existence as a profession; others indignantly repudiate this base insinuation, and point out that nursing extends back to the time of Rachel, the patriarch Jacob's wife. What is nursing? It is usually considered to be a feminine instinct, an outcome of the maternal instinct which bids a woman seek to ease pain or suffering in whatever form it is manifested. The instinct is as old as creation, its evolution through the ages full of interest. One thing that stands out prominently in its history is that we are tracing the development of an instinct wholly good and unselfish, capable, therefore, of infinite expansion. We might naturally expect to see a position attained by those who possess this instinct, commensurate with the progress attained by mankind in other directions. With every advance made by civilisation has come a corresponding need for the nursing instinct, and yet it is possible for it to be said to-day that nursing as a profession has only recently come into existence. It would be interesting to trace the causes underlying the failure of mankind, given the best material and an ever-increasing opportunity, to use these in its own interests, as it has used so many other primitive instincts; but that is not within the scope of these articles, which are more concerned with the present and the future than with the past.

From mediæval times women have been in the habit of banding themselves together in Sisterhoods or other organisations for the purpose of ministering to the sick. At one time, before the dissolution of the monasteries, the Church had the main control of nursing in England, as it still has to a large extent in Ireland, and as long as this was so nursing was as much a vocation as the more confined forms of the religious life. Gradually, during the past two or three centuries, it has evolved from a vocation into a career and a profession, which women have followed from various motives—love of humanity, the desire to "do something," the need of earning a livelihood, the same sheer interest in the healing art which attracts many men to the medical profession, and so forth. With evolution came also a good deal of evil. The idea of gain overrode all higher conceptions, and civilisation was dishonoured by women who, caring little or nothing for the individuals whose sufferings paid them their wages, used the helplessness of their patients as an excuse for cruelty and neglect. The two poles of this axis were thus far apart. At one end was the selfless devotion of the woman whose one though[illegible] was service, and to whom, therefore, payment was immaterial; at the other was the sordid outlook of the woman who would undertake for a trifling sum a work which she scarcely even attempted to do. Then came the Crimean War and the work of Florence Nightingale, herself an epitome of what a nurse should be. But in spite of the impetus thus given to nursing, and the object lesson afforded of what was needed and what could be done, development of the organism as a whole remained stunted. Not for lack of opportunity or lack of zeal. The great hospitals realised what it would mean to have their own staffs of nurses, and began to establish training-schools and to seek to attract the best type of woman, and to give her as far as possible advantages in the way of lectures and classes, and increased facilities for recreation. Nursing institutions arose, which housed and fed a staff of trained nurses, and undertook to supply the public with trained nurses in their own homes. Nurses' societies were formed for the purpose of giving fuller life to nurses, of encouraging friendly intercourse and the exchange of ideas. And here and there large ideas germinated in the minds of women—ideas which might have borne much fruit had it not been for the thorns which sprang up and choked them. But in spite of all this the public remained uninterested and apathetic. Why? Because the nursing profession resembled branches without any trunk. To ask the public to occupy itself with a mere bough, or with several boughs, at once, would be on the face of it unreasonable, however perfect in form, and graceful in varying foliage, those several boughs might be. Until there is a trunk, acting as a cohesive to the different branches and co-ordinating them in their due relations, there is nothing upon which attention can be focussed. The second decade of the twentieth century saw the nursing profession in this position, containing the highest possibilities and the lowest, but lacking the cohesion which would, while not necessarily eliminating the lowest, yet render the highest so all-persuasive that the lowest could be disregarded as a negligible quantity. Then the College was born.

